# Taxonomic Diversity and Clinical Correlations in Periapical Lesions by Next-Generation Sequencing Analysis

**DOI:** 10.3390/genes16070775

**Published:** 2025-06-30

**Authors:** Juliana D. Bronzato, Brenda P. F. A. Gomes, Tsute Chen

**Affiliations:** 1 Piracicaba Dental School, State University of Campinas-UNICAMP, Piracicaba 13083-970, SP, Brazil; julianadelatorre_@hotmail.com; 2 The ADA Forsyth Institute, Somerville, MA 02143, USA

**Keywords:** apical periodontitis, microbiota, endodontics, bioinformatics, high-throughput nucleotide sequencing

## Abstract

**Objectives:** The aim of this study was to assess the taxonomic diversity of the microbiota associated with periapical lesions of endodontic origin and to determine whether microbial profiles vary across different populations and clinical characteristics using a unified in silico analysis of next-generation sequencing (NGS) data. **Methods:** Raw 16S rRNA sequencing data from three published studies were retrieved from the NCBI Sequence Read Archive and reprocessed using a standardized bioinformatics pipeline. Amplicon sequence variants were inferred using DADA2, and taxonomic assignments were performed using BLASTN against a curated 16S rRNA reference database. Alpha and beta diversity analyses were conducted using QIIME 2 and R, and differential abundance was assessed with ANCOM-BC2. Statistical comparisons were made based on population, sex, symptomatology, and other clinical metadata. **Results:** A total of 38 periapical lesion samples yielded 566,223 high-confidence reads assigned to 347 bacterial species. Significant differences in microbial composition were observed between geographic regions (China vs. Spain), sexes, and symptoms. Core species such as *Fretibacterium* sp. HMT 360 and *Porphyromonas endodontalis* were prevalent across datasets. *Porphyromonas gingivalis* and *Fusobacterium nucleatum* were found in abundance across all three studies. Beta diversity metrics revealed distinct clustering by study and country. Symptomatic lesions were associated with higher abundance of *Alloprevotella tannerae* and *Prevotella oris*. **Conclusions:** The periapical lesion microbiota is taxonomically diverse and varies significantly by geographic and clinical features.

## 1. Introduction

A periapical lesion is a pathological alteration located around the apex of a tooth root, typically resulting from the spread of microorganisms and their byproducts from an infected root canal system into the periapical tissues [1,2]. It represents a host immune response to microbial invasion and is most commonly associated with apical periodontitis. Apical periodontitis is characterized mainly by a periapical lesion that is visualized as a radiolucent area attached to the tooth apex, seen through imaging exams [2,3], and 52% of the worldwide population is affected by this disease [4].

Periapical lesions may present histologically as granulomas, radicular cysts, or abscesses and can range from asymptomatic radiolucencies to painful inflammatory conditions [2]. It was thought that periapical lesions were sterile, that the cementum acted as an effective barrier against microorganisms, or that the bacteria were killed by the host once they reached the perirradicular tissues. However, over the years, microorganisms have been detected in periapical lesions through different methods [5].

The recent introduction of nucleic acid sequencing instruments capable of producing millions of DNA sequences read in a single run has rapidly changed the universe of microbial genetics, enabling the detection of the presence of all microorganisms present in a sample [6,7]. Next-generation sequencing (NGS) appears to be an ideal methodology for characterizing the endodontic microbiota, as it is extremely sensitive, much more so than the technologies that preceded it, such as the Sanger method and pyrosequencing [8].

The characterization of the periapical lesion microbiota is critical for understanding periapical infection and potential therapies. Previous research [9,10,11] used next-generation sequencing to sequence periapical lesions of endodontic origin. However, their sample size is small, and they targeted a specific population.

Hence, this study aimed to collect all data available from various studies on next-generation sequencing of periapical lesions of endodontic origin and analyze them in silico using bioinformatics tools to determine taxonomic diversity. The null hypothesis is that there are no variations in the microbiome of lesions from different populations or samples with varied clinical characteristics.

## 2. Methods

### 2.1. Search for Available Sequences in Databases

The NCBI SRA database (https://www.ncbi.nlm.nih.gov/sra accessed on 24 January 2024) was used to search for genetic sequences of the periapical lesions microbiota. After, the accession numbers were searched in previously published papers.

Also, to identify studies that evaluated periapical lesions from endodontic origins through next-generation sequencing methodology, 5 electronic databases (PubMed, Embase, Scopus, Web of Science, and the Cochrane Library) were searched using the following keywords: [(“next-generation sequencing”) AND (apical periodontitis OR periapical lesion OR periradicular lesion OR extraradicular lesion OR endodontic lesion OR apical granuloma OR periapical granuloma OR apical cyst OR periapical cyst)]. In addition, the grey literature was searched by using Google Scholar (the first 100 hits) and the ProQuest Dissertations and Theses database. The reference lists of selected studies were also hand-searched to identify additional relevant studies. The date of the search was February 2025.

Studies that met the inclusion criteria were included in this research. The inclusion criteria were human periapical lesions from endodontic origin, collected during periapical surgery, sequenced using next-generation sequencing technology, registered in the NCBI SRA database, and limited to datasets generated from 16S rRNA gene amplicon sequencing.

The raw sequence data were obtained in FASTQ format. Each sequence was given a sample ID for this study (Table S1). Sample metadata was gathered with the purpose of further statistical analysis (e.g., country, symptomatology, age, sex, teeth localization, and number of root canal treatments).

### 2.2. DADA2 Read Processing

The analysis utilized the DADA2 software package for correcting errors in Illumina-sequenced amplicons (https://benjjneb.github.io/dada2/index.html accessed on 2 February 2024). The DADA2 pipeline involved several steps for read quality control and analysis. These include trimming poor-quality ends, learning error rates specific to each dataset, inferring amplicon sequence variants (ASVs), merging paired reads, and removing chimeras. Through these steps, DADA2 achieved accurate sequence inference while minimizing artifacts [12].

For the trimming, an empirical approach was used to test many combinations of different trim lengths in order to achieve the best final amplicon sequence variants (ASVs). First, a random subset of each sample was created, consisting of 5000 R1 and 5000 R2 (to reduce computation time). Second, 10 bases were trimmed at a time from the ends of both R1 and R2 up to 50 bases. Third, for each combination of trimmed lengths (e.g., 300 × 300, 300 × 290, 290 × 290, etc.), the trimmed reads were subject to the entire DADA2 pipeline for chimera-filtered merged ASVs. Finally, the combination with the highest percentage of the input reads becoming final ASVs was selected for the complete set of data.

Although all studies targeted the V3–V4 region of the 16S rRNA gene, the DADA2 pipeline was run separately for each included study because the sequencing platforms differed and batch effects could arise. After DADA2, the ASV count tables of individual studies were combined for further analysis.

### 2.3. Taxonomy Assignment

Taxonomy was determined with a species-level assignment algorithm by Chen et al. [13] version 20221029 (https://github.com/tsute/FOMC_16S_rRNA_Taxonomy_Assignment_Algorithm accessed on 2 February 2024). ASV read sequences were BLASTN-searched against a combined set of 16S rRNA reference sequences. It consists of MOMD (version 5.1 https://momd.org/ftp/16S_rRNA_refseq/MOMD_16S_rRNA_RefSeq/V5.1/ accessed on 2 February 2024), the HOMD (version 15.22 https://www.homd.org/ftp/16S_rRNA_refseq/HOMD_16S_rRNA_RefSeq/V15.22/ accessed on 2 February 2024), and the NCBI 16S rRNA reference sequence set (https://ftp.ncbi.nlm.nih.gov/blast/db/16S_ribosomal_RNA.tar.gz accessed on 29 October 2022). These sequences were screened and combined to remove short sequences (<1000 nt), chimera, duplicates, and sub-sequences, as well as sequences with poor taxonomy annotation (e.g., without species information).

The NCBI BLASTN version 2.7.1+ was used with the default parameters. Reads with ≥98% sequence identity to the matched reference and ≥90% alignment length (i.e., ≥90% of the read length that was aligned to the reference and was used to calculate the sequence percent identity) were classified based on the taxonomy of the reference sequence with the highest sequence identity. If multiple reference sequences matched based on their reads, with equal percent identity and alignment length, and represented more than one species, the read was subject to chimera checking with the USEARCH program version v8.1.1861 [14]. Non-chimeric reads with multi-species best hits were considered valid and were assigned a unique species notation (i.e., spp) denoting unresolvable multiple species. The species names of these unresolved multiple species hit are available in a separate table (Table S2).

Unassigned reads (i.e., reads with <98% identity or <90% alignment length) were pooled together, and reads <200 bases were removed. The remaining reads were subject to de novo operational taxonomy unit (OTU) calling and chimera checking using the USEARCH program version v8.1.1861 [14]. The de novo OTU calling and chimera checking were performed using 98% as the sequence identity cutoff, i.e., the species-level OTU. The output of this step produced species-level de novo clustered OTUs with 98% identity. Representative reads from each of the OTUs/species were then BLASTN-searched against the same reference sequence set again to determine the closest species for these potential novel species. These potential novel species were pooled together with the reads that were assigned to the species level in the previous step for downstream analyses [15]. The read taxonomy assignment procedure is depicted in Figure S1. Heatmap profiles and abundance graphs were plotted using QIIME 2.

### 2.4. Alpha Diversity

Alpha diversity measurements were performed using three indices, Observed, Shannon, and Simpson [16], using “microbiome” package in R. After, to test whether the alpha diversity among different comparison groups is different statistically, a Kruskal–Wallis H test was used, provided by the “alpha-group-significance” function in the QIIME 2 “diversity” package.

### 2.5. Beta Diversity

For the plots, the “microbiome” package in R was used. Species profiles of the samples were first compared with the Bray–Curtis dissimilarity, which is based on the count data type. The pairwise Bray–Curtis dissimilarity matrix of all samples was then subjected to either multi-dimensional scaling (MDS, also known as PCoA) or non-metric MDS (NMDS).

The PCoA and NMDS plots are based on count data. The count data were also transformed into centered log ratio (CLR) for each species and compared with Euclidean distances (also called Aitchison distance).

To test whether the between-group dissimilarities are significantly greater than the within-group dissimilarities, the “beta-group-significance” function provided in the QIIME 2 “diversity” package was used with PERMANOVA (permutational multivariate analysis of variance) as the group significant testing method. Three beta diversity metrics were used: (1) Bray–Curtis dissimilarity, (2) Correlation coefficient matrix, and (3) Aitchison distance (Euclidean distance between CLR-transformed compositions).

### 2.6. Differential Abundance (ANCOM-BC2)

To verify differential abundance (DA) between groups, the ANCOM-BC2 (Analysis of Composition of Microbiomes with Bias Correction 2) package was used in R. The read count data was transformed into log ratio data. The ratios were calculated between read counts of all species in a sample to a “reference” count (e.g., mean read count of the sample). The log ratio data allowed for the detection of DA species without being affected by percentage bias. The bias correction (BC) estimated the unknown sampling fractions and corrected the bias induced by their differences among samples. The absolute abundance data were modeled using a linear regression framework. When performing the pairwise directional test, the mixed directional false discover rate (mdFDR) was taken into account [17].

## 3. Results

A total of three studies were included [9,10,11]. Table 1 shows the sample meta information collected and used for the statistical analysis. “Unknown” refers to the fact that it was not described in the respective paper or the SRA register.

The raw sequencing data included 771,878 reads for Zhang et al. [11], 353,875 reads for Perez-Carrasco et al. [10], and 198,448 reads for Arias-Moliz et al. [9]. The trim length combination of R1 = 251 bases and R2 = 251 bases was chosen for generating the final ASVs for the sequences in Perez-Carrasco et al. [10] and Arias-Moliz et al. [9]. The trim length combination of R1 = 240 bases and R2 = 240 bases was chosen for generating the final ASVs for the sequences in Zhang et al. [11]. This combination generated the highest number of merged non-chimeric ASVs and was used for the downstream analyses. After quality filtering, denoising, merging, and chimera removal, the final non-chimeric reads retained were 33.3% (256,657 reads) for Zhang et al. [11], 82.8% (293,018 reads) for Perez-Carrasco et al. [10], and 60.8% (120,710 reads) for Arias-Moliz et al. [9] (Table 2).

The sequencing reads from 38 samples were assigned to taxonomic groups based on BLASTN classification, with a filtering threshold (MPC = 0.01%, minimal percent of read count per species of all assigned reads) applied to remove species with low read counts. This filtering step ensured that only high-confidence taxonomic assignments were included in downstream microbiome diversity analyses. The total number of reads analyzed was 670,385, of which 571,895 (85.3%) were successfully assigned to species.

After MPC filtering, the assigned reads were 566,223 and a total of 347 species (Table S2) were retained for further analysis, of which 266 were single-species assignments, 67 were multi-species assignments, and 14 were classified as novel species. Reads that could not be assigned to any known species accounted for 98,490 reads (14.7%), consisting primarily of sequences without BLASTN hits (42,360 reads), low-quality sequences, singletons, or chimeric reads.

**Figure 1:**
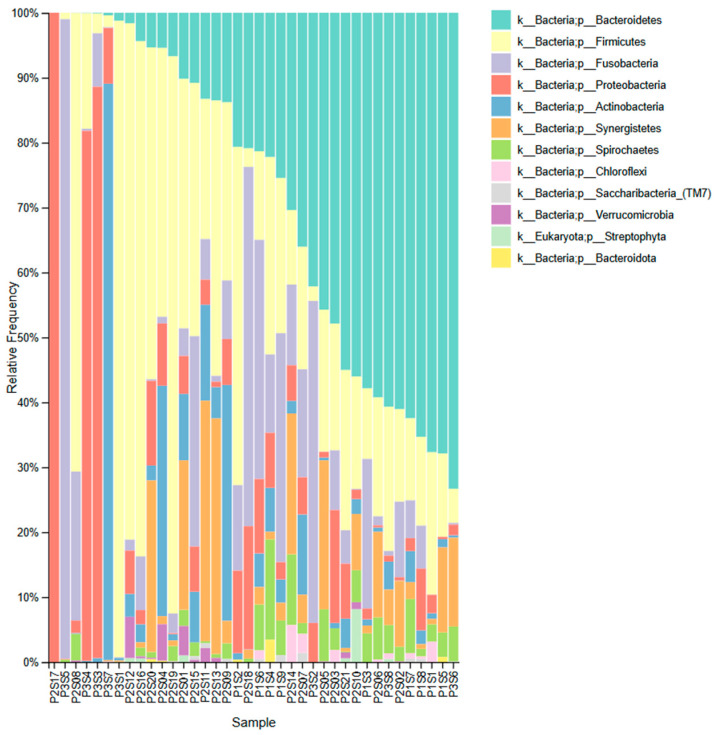
Taxonomic composition of periapical lesion samples at the phylum level across different samples. The stacked bar plot represents the relative frequency of bacterial and eukaryotic phyla detected in each sample. Each color corresponds to a different phylum, as indicated in the legend on the right (k = Kingdom, p = phylum).

The most abundant phyla across samples include Bacteroidetes, Firmicutes, Proteobacteria, Fusobacteria, and Actinobacteria, with additional contributions from Synergistetes, Spirochaetes, Chloroflexi, Saccharibacteria (TM7), Verrucomicrobia, and Streptophyta (Eukaryota) (Figure 1).

**Figure 2:**
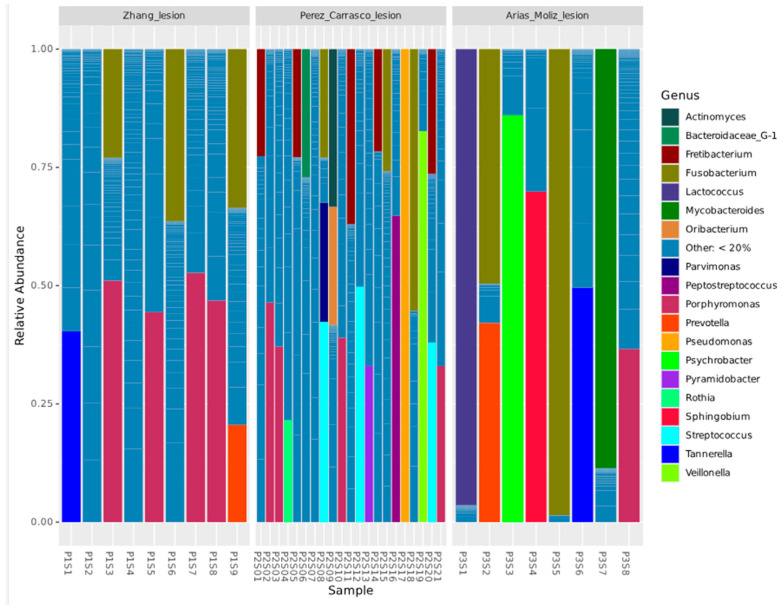
Relative abundance of the top 19 bacterial genera identified in lesion samples from different studies.

The relative abundance of the top 19 bacterial genera can be visualized through stacked bar plots, illustrating the variability in microbial profiles across samples (Figure 2). In Zhang et al. [11], a predominance of a few bacterial genera was observed. *Tannerella*, *Porphyromonas*, *Fusobacterium*, and *Prevotella* were among the most abundant genera. Conversely, Perez-Carrasco et al. [10] exhibited a highly heterogeneous microbial composition, with a greater number of genera contributing to the overall diversity. This group demonstrated a more even distribution of bacterial genera, including *Porphyromonas*, *Fretibacterium*, *Pseudomonas*, *Fusobacterium*, *Streptococcus*, *Peptostreptococcus*, and *Parvimonas*, among others. Arias-Moliz et al. [9] displayed a microbial profile with intermediate diversity between the other two groups. Certain genera, such as *Mycobacteroides*, *Porphyromonas*, *Tanerella*, *Prevotella*, and *Fusobacterium*, were present in notable proportions.

The number of species per sample varied from 1 to 166 (Table S2, Figure S2). The species found in most samples were *F.* sp. HMT 360 and *P. endodontalis* (n = 24), followed by *Treponema socranskii* and *Peptostreptococcus stomatis* (n = 22); *Dialister invisus*, *Treponema maltophilum*, and *Tannerela forsythia* (n = 21); *Parvimonas micra* (n = 20); *Haemophilus parainfluenzae*, *Fretibacterium fastidiosum*, and *Campylobacter* sp. (n = 19); *Veillonela dispar* (n = 18); *Granilucatella adiacens* (n = 17); *F. nucleatum* subsp. *vicentii* (n = 16); *Slackia exigua*, *P. oris*, and *Treponema denticola* (n = 15); and *Filifactor alocis* (n = 14).

**Figure 3:**
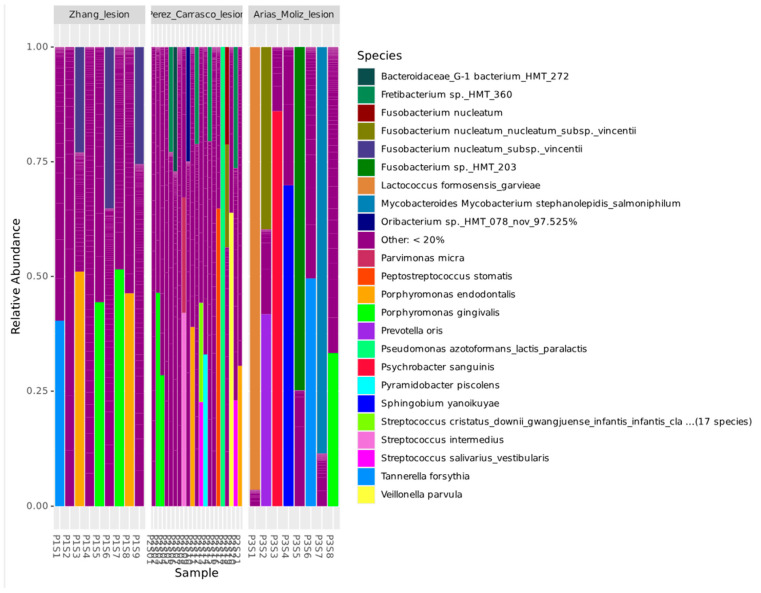
Relative abundance of the top 23 bacterial species identified in lesion samples from different studies.

The microbial composition of lesion samples was analyzed at the species level, revealing distinct taxonomic profiles among the three study groups (Figure 3). In the Zhang et al. [11] group, the microbial composition was dominated by *P. gingivalis*, *P. endodontalis*, *T. forsythia*, and *F. nucleatum*. In contrast, among the most abundant species in Perez-Carrasco et al. [10] were *F.* sp. HMT 360, *Pseudomonas azotoformans*, *F. nucleatum*, *P. stomatis*, *P. micra*, *Veillonella parvula*, *P. endodontalis*, *Streptococcus intermedius*, *Streptococcus salivarius*, and *P. gingivalis*. In Arias-Moliz et al. [9], species such as *Mycobacteroides stephanolepidis*, *P. gingivalis*, *T. forsythia*, *F. nucleatum*, *Fusobacterium* sp. HMT 203, *Sphingobium yanoikuyae*, *Psychrobacter sanguinis*, *P. oris*, and *Lactococcus formosensis* were identified in high abundance.

**Figure 4:**
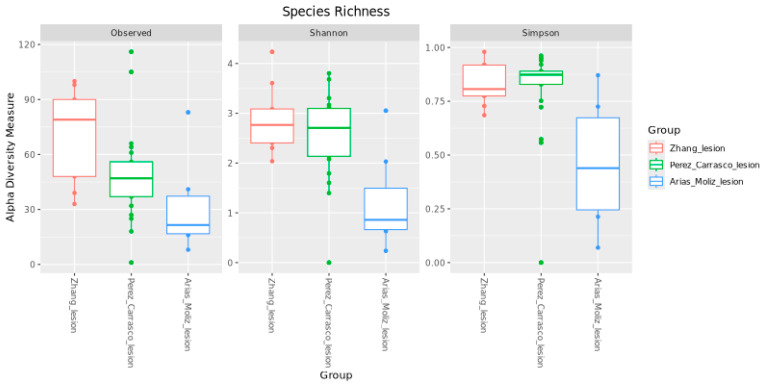
Alpha diversity measures of the microbiota in periapical lesions from three published studies.

**Figure 5:**
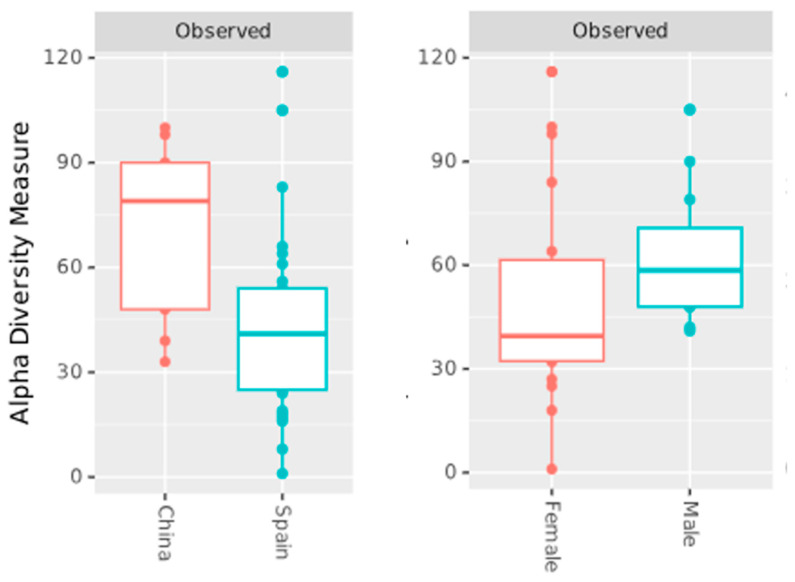
Observed species richness in periapical lesion microbiota based on geographic origin and sex.

Regarding the alpha diversity (Figure 4), the Observed index revealed that the Zhang et al. [11] and Perez-Carrasco et al. [10] groups exhibited higher species richness compared to the Arias-Moliz et al. [9] group. The Shannon index, which accounts for both richness and evenness, and the Simpson index, a measure of evenness, showed a similar trend. There was a statistically significant difference between Arias-Moliz et al. [9] when compared to Zhang et al. [11] (Observed, q = 0.007; Shannon, q = 0.004; Simpson, q = 0.005) and Perez-Carrasco et al. [10] (Observed, q = 0.03; Shannon, q = 0.004; Simpson, q = 0.002). No significant differences were observed between the Zhang et al. [11] and Perez-Carrasco et al. [10] datasets for any of the diversity indices. When other groups were analyzed, a statistically significant difference was found in the Observed index between the China and Spain groups (q = 0.01), and female and male groups (q = 0.046) (Figure 5). No difference was found between the symptomatic and asymptomatic groups.

**Figure 6:**
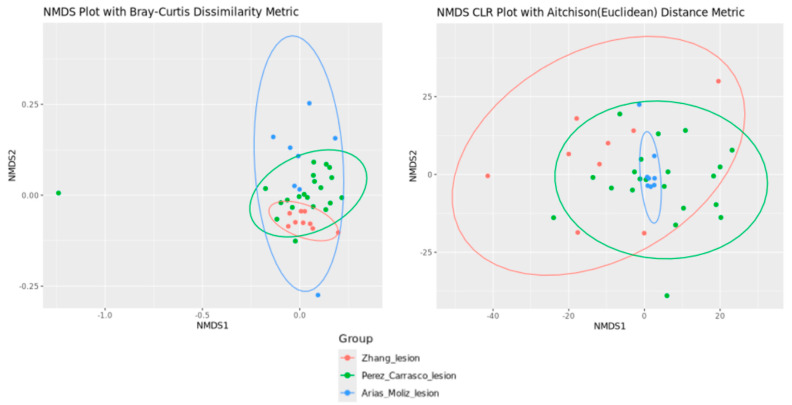
Beta diversity analysis of the microbiota in periapical lesions of the three included studies using non-metric multi-dimensional scaling (NMDS) with the Bray—Curtis dissimilarity metric or Aitchison distance metric.

**Figure 7:**
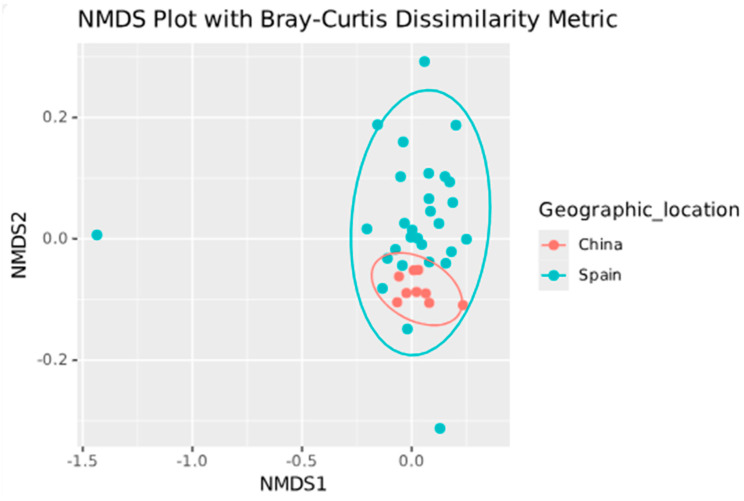
Beta diversity analysis of the microbiota in periapical lesions by geographic location using non-metric multi-dimensional scaling (NMDS) with the Bray—Curtis dissimilarity metric.

Regarding beta diversity (Figure 6 and Figure 7), significant differences were found between the Zhang et al. [11] and Perez-Carrasco et al. [10] groups using the Bray–Curtis dissimilarity (q = 0.001) and Aitchison distance (q = 0.001) metrics. A significant difference was also observed between the Zhang et al. [11] and Arias-Moliz et al. [9] groups using the Bray–Curtis dissimilarity (q = 0.004) and Aitchison distance (q = 0.007) metrics. No significant difference was found between the Arias-Moliz et al. [9] and Perez-Carrasco et al. [10] groups; however, a significant difference was detected between the China and Spain groups using the Bray–Curtis dissimilarity (q = 0.001) and Aitchison distance (q = 0.001) metrics.

According to the ANCOM-BC2 differential abundance analysis, *Fretibacterium* sp HMT 359 (SP222) and *Pseudoramibacter alactolyticus* (SP672) were more abundant in Spanish samples than in Chinese (q < 0.05). On the other hand, the *Lachnospiraceae* [G-8] bacterium HMT 500 (SP29) was more abundant in the Chinese population than in the Spanish population (q < 0.05). *A. tannerae* (SP43) and *P. oris* (SP35) were more abundant in symptomatic than asymptomatic samples (q < 0.05). *Peptostreptococcaceae* [XI] [G-4] *bacterium* HMT 369 (SP497), *Selenomonas* sp. HMT 134 (SP19) and *Gemella morbillorum* (SP109) were more abundant in male samples than female samples (q < 0.05).

## 4. Discussion

This study combined and reanalyzed 16S rRNA NGS datasets from three independent studies [9,10,11] to provide a comprehensive analysis of the microbiota found in human periapical lesions. By combining raw sequencing data with a unified bioinformatics pipeline, it was possible to compare microbial communities from various geographic locations and clinical contexts, providing new insights into the diversity of the periapical lesion microbiome. The findings rejected the null hypothesis, confirming that the microbiome of lesions varies by population and clinical profile.

The predominant phyla identified across samples (Bacteroidetes, Firmicutes, Proteobacteria, Fusobacteria, and Actinobacteria) are consistent with those reported in endodontic infections and periodontal sites [18]. At the genus level, *Porphyromonas*, *Tannerella*, *Fusobacterium*, and *Prevotella* were frequently abundant, consistent with their known roles as anaerobic pathogens in periradicular lesions [5,19]. *F.* sp. *HMT 360* and *P. endodontalis* were the most frequently detected species (63%), indicating the presence of a potential core microbiome in apical lesions despite inter-study heterogeneity. *P. endodontalis* was detected in periapical lesions at a similar rate (62.5%) in the literature [19] using Nested-PCR. Furthermore, *P. endodontalis* and *Fretibacterium* sp. are frequently found in both primary and secondary infections in teeth with apical periodontitis [20,21,22].

*P. gingivalis* and *F. nucleatum* were found in abundance across all three studies [9,10,11]. These are well-known periodontal pathogens [23], and their high abundance in periapical lesions across studies suggests a common microbial signature between periodontal and endodontic infections. This lends support to the concept of functional redundancy in microbial pathogenesis, which was also emphasized by Arias-Moliz et al. [9], in which different microbial consortia can exert similar pathogenic effects.

*P. gingivalis* is a periodontal pathogen known for its ability to evade the host’s immune response. Furthermore, *P. gingivalis* can use Toll-like receptor signaling and neutrophil function to its advantage, facilitating chronic infection [24]. *F. nucleatum*, on the other hand, is regarded as a key bridging organism in multispecies biofilms due to its ability to coaggregate with both early and late colonizers, such as *Streptococcus*, *Prevotella*, and *Porphyromonas* spp. [25]. *F. nucleatum*’s bridging capacity allows it to maintain complex microbial communities in anaerobic niches like root canals and periapical tissues [5,26,27]. It can also invade epithelial and endothelial cells and has been linked to systemic dissemination [26]. Arias-Moliz et al. [9] identified *F. nucleatum* as a keystone taxon based on its widespread distribution, centrality in co-occurrence networks, and abundance. Although our reanalysis did not explicitly compute network centrality, this species’ consistent detection and abundance highlight its ecological and pathogenic importance.

A key methodological strength of the present study is the species-level resolution achieved through reanalysis of raw sequencing data using a unified pipeline. Only Arias-Moliz et al. [9] conducted a species-level analysis, whereas Zhang et al. [11] and Pérez-Carrasco et al. [10] reported a microbial composition up to the genus level. By reclassifying sequences using a curated reference database with strict identity thresholds, our study allowed for more precise taxonomic assignment and identification of specific species associated with geographic or clinical variables. This high-resolution approach improved the interpretability of microbial profiles by detecting up to 166 species per sample. The higher microbial diversity indicates a complex and variable microbiota within these lesion samples, which is consistent with the literature [5]. Overall, the microbial composition at the species level varied significantly between the three studies. The microbiota in Zhang et al. [11] samples was highly dominant, while Perez-Carrasco et al. [10] samples had the highest species diversity, and Arias-Moliz et al. [9] samples had an intermediate microbial structure. These findings suggest that disease progression, host responses, and environmental conditions may all have an impact on bacterial community variation. The discovery of 14 potential novel species highlights the unexplored diversity of periapical lesions. Although their functional roles are unknown, their occurrence in multiple samples suggests that they may contribute to the chronic apical infections’ polymicrobial ecosystems.

The uncertainty of microbial viability is an important factor to consider in studies that use DNA-based sequencing. The presence of microbial DNA does not guarantee that the organisms are alive or metabolically active [28]. Some of the microorganisms detected could be remnants of previously active biofilms, or they could be present only temporarily due to hematogenous spread or contamination. Previous meta-analyses, however, have found that up to 82% of periapical lesions contain viable or active microorganisms when culture or RNA-based methods are used [5,19]. Furthermore, a previous study found bacteria embedded in periapical tissues [29], and the identification of keystone species in recent network analyses [9] suggests that some bacteria are more than just passing through, playing an active ecological and potentially pathogenic role. Many of the detected taxa, such as *F. nucleatum*, *P. endodontalis*, and *T. forsythia*, are obligate anaerobes known to form structured biofilms, which can protect them from host immune responses and create microenvironments that sustain long-term survival, consistent with previous culture-based and molecular evidence indicating that periapical lesions serve as a stable reservoir for anaerobic bacteria [5,19]. Low oxygen tension, the availability of necrotic tissue as a nutrient source, and impaired local immune clearance may all contribute to the survival of viable anaerobic microbiota in periapical lesions.

The results revealed the presence of Eukarya in five samples from Pérez-Carrasco et al. [10] and one from Arias-Moliz et al. [9], which was an unexpected but noteworthy finding. While next-generation sequencing of 16S rRNA genes is intended to target prokaryotes, the universal primers used may occasionally amplify chloroplast or mitochondrial 16S-like sequences from plant cells, which have evolutionary ancestry with bacterial ribosomes [30]. The presence of plant DNA in human periapical samples could be attributed to dietary residue contamination, and certain plant sequences may also be present as environmental contaminants in reagents or instruments [31]. Although these sequences were not common, their detection emphasizes the importance of strict contamination controls and careful interpretation of non-bacterial sequences in clinical microbiome studies.

Significant differences in alpha diversity were found across datasets, with the Spanish studies [9,10] exhibiting lower richness in some comparisons. This finding could be attributed to the study of Arias-Moliz et al. [9], which found the lowest richness and evenness. However, these differences were not always associated with the presence or absence of clinical symptoms, which is consistent with Pérez-Carrasco et al. [10], who discovered only minor taxonomic differences between symptomatic and asymptomatic lesions. The lack of diversity differences between symptomatic and asymptomatic groups implies that microbial richness alone may not predict clinical symptoms. The present study expands on these findings by identifying a few species with significantly higher abundance in symptomatic lesions (*A. tannerae* and *P. oris*), implying that specific species may contribute disproportionately to clinical manifestations.

*P. oris* is a Gram-negative anaerobe commonly found in endodontic infections [32,33]. The presence of *P. oris* antibiotic resistance genes may explain its presence in persistent endodontic infections [32]. Similarly, *A. tannerae* (formerly *Prevotella tannerae*) has been detected in endodontic infections [34], which may explain its presence in periapical lesions. These species have numerous virulence factors that could contribute to the symptomatology of the cases [35,36], implying that they may participate in the exacerbation of host responses, pain, or swelling in periapical infections.

The beta diversity analysis revealed consistent differences among samples from various geographical origins. The Chinese samples [11] had distinct microbial profiles from the Spanish datasets, a finding that was consistent across the Bray–Curtis and Aitchison metrics. This was consistent with the analysis which found that Perez-Carrasco et al. [10] and Arias-Moliz et al. [9] had similar beta diversity levels. This is likely due to population-specific factors such as diet, genetics, and healthcare practices, which align with recent research demonstrating geographic variation in oral microbiomes [5,37]. The differential abundance of species such as *P. alactolyticus* and *F.* sp. HMT 359 (more abundant in Spain) and *Lachnospiraceae* [G-8] *bacterium* (more abundant in China) supports the existence of biogeographic signatures in periapical lesions. Furthermore, sex-based differences were found in both alpha and differential abundance metrics, with certain taxa (such as *G. morbillorum*) being more abundant in male patients. While the underlying mechanisms are unclear, hormonal or immunological differences may influence microbial colonization or persistence [38].

All three studies obtained periapical lesion samples through endodontic microsurgery rather than extracted teeth, which is a significant strength. This sampling strategy reduces contamination from the oral cavity or root canal system and allows for more direct access to infected periapical tissues, increasing the reliability of the detected microbiota [19,39]. However, the sample processing protocols and sequencing platforms used in the included studies differed significantly, which could have an impact on microbiota recovery and diversity metrics. Zhang et al. [11] stored periapical samples in phosphate-buffered saline (PBS) at the time of collection, whereas Pérez-Carrasco et al. [10] and Arias-Moliz et al. [9] cryo-pulverized the tissues before DNA extraction. Cryo-pulverization is likely to improve lysis efficiency and nucleic acid yield from dense or calcified tissue matrices, potentially allowing for the detection of deeper, tissue-embedded microbes [8]. Furthermore, sequencing platforms differed: Zhang et al. used the Illumina NovaSeq 6000, which has significantly higher throughput and read depth than the MiSeq platforms used in the other two studies [7,40]. While deeper sequencing can improve the detection of low-abundance taxa, it may also introduce more noise or background contamination if not carefully controlled [7,40].

These methodological differences may account for some of the observed variation in alpha diversity and taxonomic profiles across studies, as well as the variation in final read retention. Nonetheless, our standardized bioinformatic pipeline and rarefaction strategy helped to reduce these differences, allowing for meaningful cross-study comparisons at the species level. Methodologically, by applying consistent filtering, taxonomic assignment, and diversity analyses to raw sequencing data, it was possible to overcome some of the limitations of study-specific pipelines and improve statistical robustness. However, it is important to note that differences in DNA extraction methods, lesion sampling techniques, and sequencing platforms may still cause batch effects. Despite this, our findings were broadly consistent with each study’s original conclusions, demonstrating the validity of our approach.

The findings confirm the taxonomic diversity and complexity of the microbiota associated with periapical lesions, as well as the presence of distinct microbial signatures related to geography and clinical parameters. The discovery of recurrent species, including potential keystone taxa, emphasizes the importance of more targeted microbiological diagnostics and treatment strategies. The results offer insights that could eventually enhance clinical decision-making in endodontics, particularly by identifying bacterial species associated with symptomatic or persistent periapical lesions. Such knowledge could help tailor therapeutic approaches, guide retreatment decisions, or inform prognosis. However, applying these findings in real-world settings remains challenging. DNA-based methods detect microbial presence but not viability or activity, and sequencing technologies are not yet practical for routine clinical use. Future research should focus on developing rapid, cost-effective diagnostic tools that can be feasibly implemented in clinical practice. Integrating high-resolution sequencing with clinical metadata holds promise for improving personalized endodontic care. Future studies using shotgun metagenomics or metatranscriptomics will be required to investigate microbial function in greater detail.

The limitations of this study include heterogeneity in sampling methods, incomplete clinical metadata, and the inability of DNA-based approaches to confirm microbial viability. In particular, the lack of standardized data on treatment history, lesion progression, and clinical signs such as fistula or sinus tract limits the ability to draw strong clinical correlations. Future studies should aim to combine microbiome profiling with detailed clinical follow-up, exploring associations with healing outcomes, treatment response, and symptomatology, including asymptomatic lesions and those presenting with fistulas.

## 5. Conclusions

The periapical lesion microbiota is taxonomically diverse and varies significantly by geographic and clinical features.

## Figures and Tables

**Table 1:** Characteristics of included studies.

Study Characteristics	Zhang et al. [11]	Perez-Carrasco et al. [10]	Arias-Moliz et al. [9]
Year	2022	2023	2024
Country	China	Spain	Spain
Journal	*Frontiers in Cellular and Infection Microbiology*	*International Endodontic Journal*	*International Endodontic Journal*
Study type	Observational	Observational	Observational
Bioproject registration	PRJNA808987	PRJNA839210	PRJNA1033443
Industrial funding	No	No	No
Number of samples analyzed in the present study	9 periapical lesions	21 periapical lesions	8 periapical lesions
Mean age (SD)	36 (13.37)	45.9 (13.9)	Unknown
Teeth type	Single and multi-rooted	Single, bi-, and multi-rooted	Unknown
Teeth localization	Upper and lower	Upper and lower	Unknown
Type of operator	Experienced endodontist	Experienced endodontist	Experienced endodontist
Number of previous root canal treatments	1 to 2	1	Unknown
Lesion collection	curette	curette and tweezers	curette and tweezers
Use of magnification	yes	yes	yes
Sample storage	Sterile PBS	Cryopulverization	Cryopulverization
Inclusion criteria	Endodontically treated teeth with adequate coronal restoration and true periapical lesions without involving the periodontal tissues (probing depth [PD] ≤3 mm).	Previously treated root canal for at least 1 year with radiographic evidence of persistent apical periodontitis.	Patients showing radiographic evidence of persistent apical periodontitis.
Exclusion criteria	History of antibiotic use in the past month, vertical root fracture, and resurgery teeth.	Severe systemic disease, use of antibiotics in the 3 months previous to surgery, teeth with periodontal pockets >4 mm, pulp cavity exposure to the oral environment, teeth with vertical root fracture, and history of trauma. Pregnant or breastfeeding women and patients under 18 years old were also excluded.	Patients with severe systemic diseases, pregnant or breastfeeding women, and patients under 18 years old. Teeth with periodontal pockets >4 mm, vertical root fracture, and history of trauma.
Illumina Sequencer	NovaSeq 6000	MiSeq	MiSeq
Targeted 16S rRNA gene region	V3–V4	V3–V4	V3–V4

**Table 2:** Summary of DADA2 processing steps for all samples of the included papers, showing the total number of reads and the percentage of reads retained at each step. The table includes the following steps: Input (raw sequence reads), Filtered (after quality filtering), DenoisedF (forward read denoising), DenoisedR (reverse read denoising), Merged (paired-end merging), and Nonchim (final non-chimeric reads used for downstream analysis). The percentage values represent the proportion of reads retained at each step relative to the initial input reads regarding all samples according to each paper.

Step	Zhang et al. [11]—Reads	Zhang et al.—%	Perez-Carrasco et al. [10]—Reads	Perez-Carrasco et al.—%	Arias-Moliz et al. [9]—Reads	Arias-Moliz et al.—%
Input	771,878	100.0	353,875	100.0	198,448	100.0
Filtered	757,227	98.1	348,361	98.4	197,833	99.7
DenoisedF	735,235	95.3	324,606	91.7	152,593	76.9
DenoisedR	724,395	93.8	317,580	89.7	142,944	72.0
Merged	702,680	91.0	297,976	84.2	122,709	61.8
Nonchim	256,657	33.3	293,018	82.8	120,710	60.8

## Data Availability

The original contributions presented in this study are included in the article/Supplementary Material. Further inquiries can be directed to the corresponding author(s).

## Supplementary Materials

The following supporting information can be downloaded: https://www.mdpi.com/article/10.3390/genes16070775/s1, Figure S1: Species–level taxonomy assignment pipeline flowchart; Figure S2: Species versus sample abundance heatmap for all samples; Table S1: Sample ID corresponding to each R1 and R2 FASTQ file included in this study; Table S2: Species detected in periapical lesion samples according to the read counts for each sample (columns) and each species identified based on the ASV sequences.
