# Supplementary material for: Taxonomic Diversity and Clinical Correlations in Periapical Lesions by Next-Generation Sequencing Analysis
Source: Genes (Basel). 2025 Jun 30;16(7):775. doi: 10.3390/genes16070775 (PMC12294204; doi:10.3390/genes16070775)

Species–Level Open Reference Taxonomy Assignment for NGS 16S rRNA Reads

Tsute Chen & Nezar Al-hebshi

Version 2022-10-29

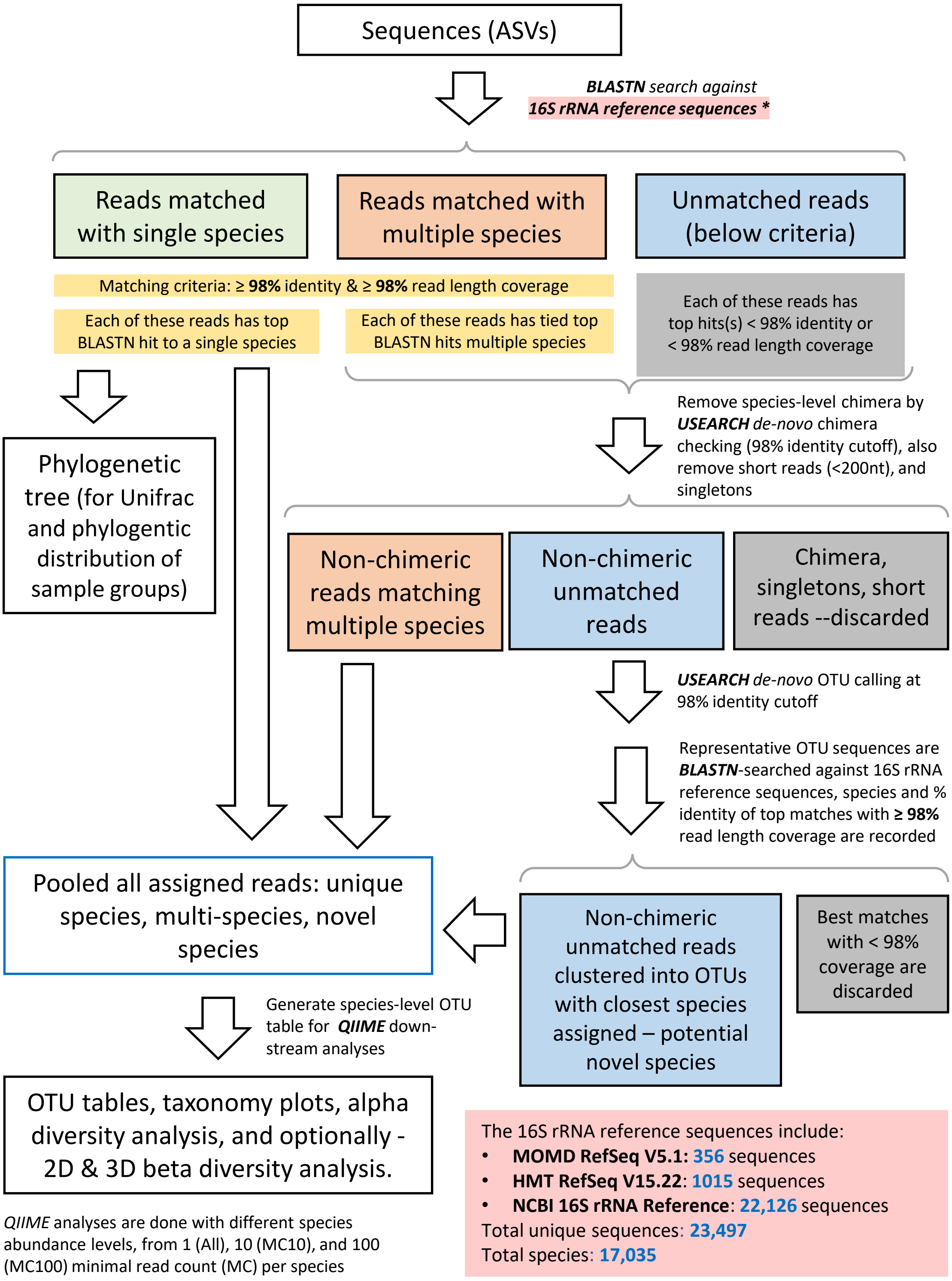

Supplement: Supplementary file 1 [file genes-16-00775-s001.zip › Figure S1 Species-Level_BALSTN-QIIME-Pipeline-Flowchart_lettersize_20221029.pdf]
