# Supplementary material for: Taxonomic Diversity and Clinical Correlations in Periapical Lesions by Next-Generation Sequencing Analysis
Source: Genes (Basel). 2025 Jun 30;16(7):775. doi: 10.3390/genes16070775 (PMC12294204; doi:10.3390/genes16070775)
Supplement: Supplementary file 1 [file genes-16-00775-s001.zip › suppl table 1.pdf]

Suppl Table S1. Sample ID corresponding to each R1 and R2 FASTQ file included in this study.

Sample ID	Read 1 File Name	R1 Read Count	Read 2 File Name	R2 Read Count	Group
P1S1	SRR18111905_1.fastq	92365	SRR18111905_2.fastq	92365	Zhang_lesion
P1S2	SRR18111913_1.fastq	96434	SRR18111913_2.fastq	96434	Zhang_lesion
P1S3	SRR18111914_1.fastq	84728	SRR18111914_2.fastq	84728	Zhang_lesion
P1S4	SRR18111915_1.fastq	82327	SRR18111915_2.fastq	82327	Zhang_lesion
P1S5	SRR18111916_1.fastq	81930	SRR18111916_2.fastq	81930	Zhang_lesion
P1S6	SRR18111917_1.fastq	74284	SRR18111917_2.fastq	74284	Zhang_lesion
P1S7	SRR18111918_1.fastq	82239	SRR18111918_2.fastq	82239	Zhang_lesion
P1S8	SRR18111919_1.fastq	87223	SRR18111919_2.fastq	87223	Zhang_lesion
P1S9	SRR18111920_1.fastq	90348	SRR18111920_2.fastq	90348	Zhang_lesion
P2S01	SRR19261783_1.fastq	3475	SRR19261783_2.fastq	3475	Perez_Carrasco_lesion
P2S02	SRR19261785_1.fastq	39453	SRR19261785_2.fastq	39453	Perez_Carrasco_lesion
P2S03	SRR19261787_1.fastq	24915	SRR19261787_2.fastq	24915	Perez_Carrasco_lesion
P2S04	SRR19261788_1.fastq	5498	SRR19261788_2.fastq	5498	Perez_Carrasco_lesion
P2S05	SRR19261791_1.fastq	27834	SRR19261791_2.fastq	27834	Perez_Carrasco_lesion
P2S06	SRR19261793_1.fastq	19984	SRR19261793_2.fastq	19984	Perez_Carrasco_lesion
P2S07	SRR19261795_1.fastq	25076	SRR19261795_2.fastq	25076	Perez_Carrasco_lesion
P2S08	SRR19261797_1.fastq	20813	SRR19261797_2.fastq	20813	Perez_Carrasco_lesion
P2S09	SRR19261799_1.fastq	36050	SRR19261799_2.fastq	36050	Perez_Carrasco_lesion
P2S10	SRR19261801_1.fastq	7274	SRR19261801_2.fastq	7274	Perez_Carrasco_lesion
P2S11	SRR19261802_1.fastq	3400	SRR19261802_2.fastq	3400	Perez_Carrasco_lesion
P2S12	SRR19261804_1.fastq	11540	SRR19261804_2.fastq	11540	Perez_Carrasco_lesion
P2S13	SRR19261806_1.fastq	5930	SRR19261806_2.fastq	5930	Perez_Carrasco_lesion
P2S14	SRR19261808_1.fastq	5432	SRR19261808_2.fastq	5432	Perez_Carrasco_lesion
P2S15	SRR19261810_1.fastq	23417	SRR19261810_2.fastq	23417	Perez_Carrasco_lesion
P2S16	SRR19261813_1.fastq	23568	SRR19261813_2.fastq	23568	Perez_Carrasco_lesion
P2S17	SRR19261815_1.fastq	33141	SRR19261815_2.fastq	33141	Perez_Carrasco_lesion

P2S18	SRR19261817_1.fastq	12311	SRR19261817_2.fastq	12311	Perez_Carrasco_lesion
P2S19	SRR19261819_1.fastq	17112	SRR19261819_2.fastq	17112	Perez_Carrasco_lesion
P2S20	SRR19261821_1.fastq	4305	SRR19261821_2.fastq	4305	Perez_Carrasco_lesion
P2S21	SRR19261823_1.fastq	3347	SRR19261823_2.fastq	3347	Perez_Carrasco_lesion
P3S1	SRR26560479_1.fastq	22654	SRR26560479_2.fastq	22654	Arias_Moliz_lesion
P3S2	SRR26560481_1.fastq	27020	SRR26560481_2.fastq	27020	Arias_Moliz_lesion
P3S3	SRR26560484_1.fastq	16881	SRR26560484_2.fastq	16881	Arias_Moliz_lesion
P3S4	SRR26560486_1.fastq	25554	SRR26560486_2.fastq	25554	Arias_Moliz_lesion
P3S5	SRR26560488_1.fastq	7099	SRR26560488_2.fastq	7099	Arias_Moliz_lesion
P3S6	SRR26560491_1.fastq	19519	SRR26560491_2.fastq	19519	Arias_Moliz_lesion
P3S7	SRR26560492_1.fastq	26028	SRR26560492_2.fastq	26028	Arias_Moliz_lesion
P3S8	SRR26560493_1.fastq	53693	SRR26560493_2.fastq	53693	Arias_Moliz_lesion
