# Supplementary figures and images for: Taxonomic Diversity and Clinical Correlations in Periapical Lesions by Next-Generation Sequencing Analysis
Source: Genes (Basel). 2025 Jun 30;16(7):775. doi: 10.3390/genes16070775 (PMC12294204; doi:10.3390/genes16070775)

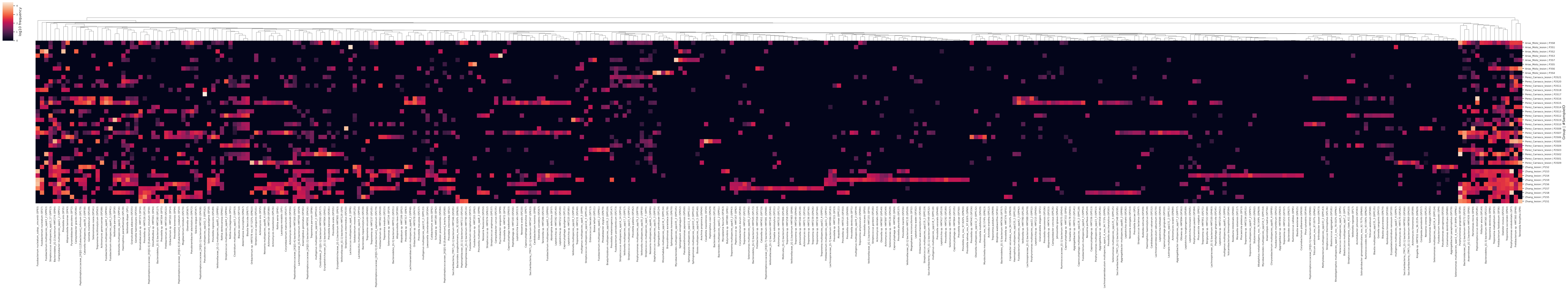

Supplement: Supplementary file 1 [file genes-16-00775-s001.zip › Figure S2-feature-table-heatmap.png]
